# Persistence of *Plasmodium falciparum* parasitemia after artemisinin combination therapy: evidence from a randomized trial in Uganda

**DOI:** 10.1038/srep26330

**Published:** 2016-05-20

**Authors:** Hsiao-Han Chang, Elamaran Meibalan, Justin Zelin, Rachel Daniels, Alice C. Eziefula, Evan C. Meyer, Fitsum Tadesse, Lynn Grignard, Regina C. Joice, Chris Drakeley, Dyann F. Wirth, Sarah K. Volkman, Caroline Buckee, Teun Bousema, Matthias Marti

**Affiliations:** 1 Center for Communicable Disease Dynamics, Department of Epidemiology, Harvard T.H. Chan School of Public Health, 665 Huntington Ave, Boston, MA 02115, USA; 2 Department of Immunology and Infectious Diseases, Harvard T.H. Chan School of Public Health, 665 Huntington Ave, Boston, MA 02115, USA; 3 Broad Institute of MIT and Harvard, 415 Main Street, Cambridge, MA 02142, USA; 4 Immunology and Infection Department, London School of Hygiene and Tropical Medicine, Keppel Street, London, WC1E 7HT, UK; 5 Simmons College, 300 Fenway, Boston, MA 02115, USA; 6 Institute for Molecular Life Sciences, Radboud University, Geert Grooteplain Zuid 28, 6525 GA Nijmegen, The Netherlands

## Abstract

Artemisinin resistance is rapidly spreading in Southeast Asia. The efficacy of artemisinin-combination therapy (ACT) continues to be excellent across Africa. We performed parasite transcriptional profiling and genotyping on samples from an antimalarial treatment trial in Uganda. We used qRT-PCR and genotyping to characterize residual circulating parasite populations after treatment with either ACT or ACT-primaquine. Transcripts suggestive of circulating ring stage parasites were present after treatment at a prevalence of >25% until at least 14 days post initiation of treatment. Greater than 98% of all ring stage parasites were cleared within the first 3 days, but subsequently persisted at low concentrations until day 14 after treatment. Genotyping demonstrated a significant decrease in multiplicity of infection within the first 2 days in both ACT and ACT-primaquine arms. However, multiple clone infections persisted until day 14 post treatment. Our data suggest the presence of genetically diverse persisting parasite populations after ACT treatment. Although we did not demonstrate clinical treatment failures after ACT and the viability and transmissibility of persisting ring stage parasites remain to be shown, these findings are of relevance for the interpretation of parasite clearance transmission dynamics and for monitoring drug effects in *Plasmodium falciparum* parasites.

Malaria remains a leading cause of global morbidity and mortality with approximately 584,000 deaths and 198 million clinical cases of malaria in 2013^1^. Prompt administration of effective antimalarial treatment remains a critical strategy for reducing the burden of malaria^2^,^3^. Artemisinin combination therapy (ACT) is universally recommended for uncomplicated *P. falciparum* malaria, and continued efficacy is crucial for successful elimination and eradication efforts. The high potency of artemisinins results in a rapid initial reduction of the biomass of asexual malaria parasites, while the more slowly eliminated partner drug contributes to parasite clearance and prevents subsequent recrudescent infections^4^,^5^. Despite the emergence and spread of parasites with a slow clearing phenotype after artesunate monotherapy across Southeast Asia, parasite clearance remains rapid in Africa with <5% of patients being parasite positive by microscopy on day 3 after initiation of ACT treatment^6^,^7^. However, at least one third of patients may remain parasite positive by DNA-based molecular detection methods^8^,^9^. If this DNA persistence reflects persistence of parasites following treatment, this may have implications for the likelihood of onward malaria transmission to mosquitoes, the feasibility of elimination in low transmission settings, and for the evolution of drug resistant parasites.

The significance of DNA-based detection of residual parasitemia after ACT treatment is currently unknown. While residual submicroscopic parasitemia has been associated with subsequent recrudescence of infections^8^, a PCR signal may also be explained by residual circulating DNA molecules, or by the persistence of non-replicating mature gametocytes that can persist at low concentrations for several weeks after treatment^8^. The persistence of replicating or dormant asexual stage parasites after ACT treatment would have important implications for understanding mechanisms of drug action and the ability of parasites to tolerate drug treatment^10^,^11^. Here, we use a recently developed transcript-based qRT-PCR assay^12^ to sensitively detect and quantify asexual and gametocyte populations at submicroscopic levels after treatment with the ACT artemether-lumefantrine alone (AL) or in combination with the gametocytocidal drug primaquine (AL−PQ).

## Results

### Persistence of RNA transcripts suggestive of circulating ring and mature gametocyte stages

**Figure 1:**
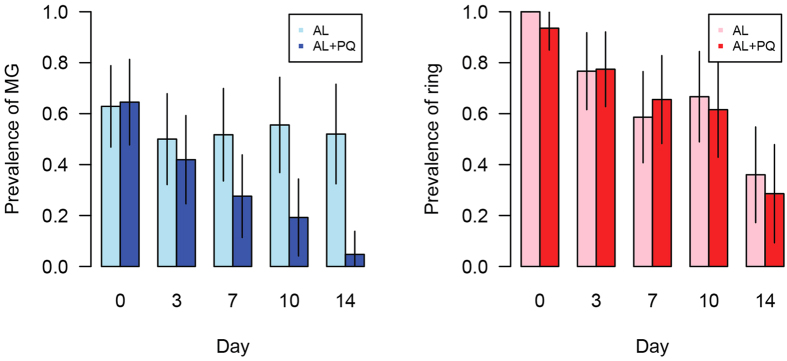
Changes in asexual and sexual stage parasite prevalence over time. **(A)** The prevalence of mature gametocytes (MG, *PF3D7_1438800*) decreases more rapidly in the AL-PQ group (the slope of simple linear regression = −0.0045 (95% CI = [−0.014, 0.0047]) for the AL group and −0.040 [95% CI = [−0.049, −0.032] for the AL + PQ group). **(B)** The prevalence of rings (*PF3D7_0501300*) through time is similar in the AL group and the AL + PQ group (the slope of simple linear regression = −0.040 (95% CI = [−0.057, −0.023]) for the AL group and −0.042 [95% CI = [−0.054, −0.029] for the AL + PQ group).

**Figure 2:**
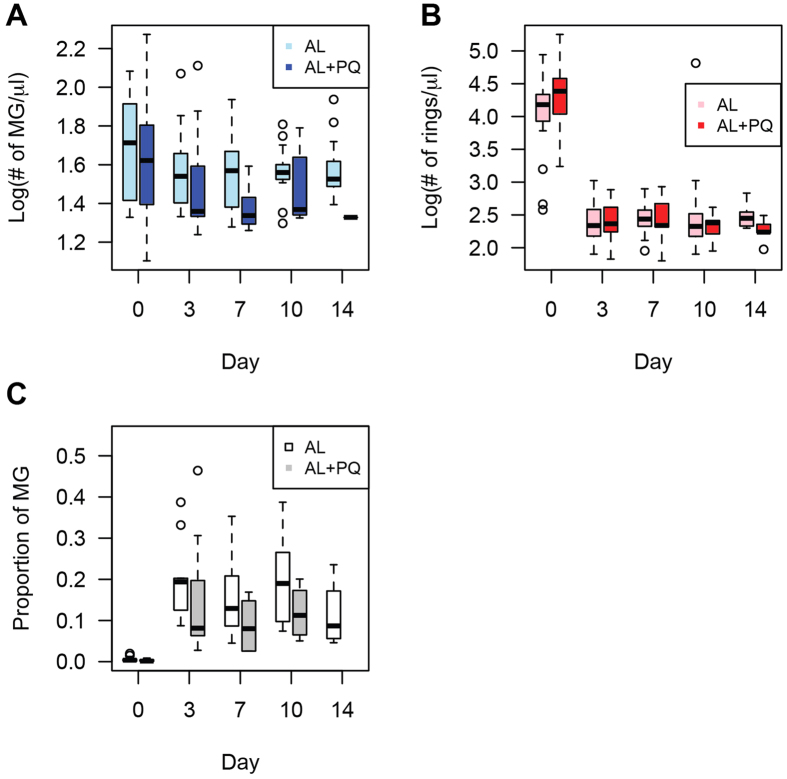
Changes in ring stage and mature gametocyte stages through time. (**A**) The number of mature gametocytes after day 3 is significantly smaller in the AL + PQ group (*p*-value = 0.0057). **(B)** The number of rings decreases between day 0 and day 3, and the level of the decrease is similar in the AL group and the AL + PQ group (*p*-value = 0.18). **(C)** The proportion of the total parasite population that represents mature gametocytes increases during follow-up (Mann-Whitney test, *p*-values = 7.92 × 10^−10^ [AL] and 1.01 × 10^−7^ [AL + PQ]).

In the original analysis of samples analyzed in the AL or AL−PQ treatment arms of the study, total parasitemia and presence of gametocytes were determined by microscopy, and *Pfs25* QT-NASBA was used for mature gametocyte detection. This analysis demonstrated complete clearance of patent parasitemia at day 3 in both treatment arms, and clearance of mature gametocyte transcripts in 94% of patients by day 14 upon primaquine treatment^13^. Using a recently developed and highly sensitive qRT-PCR assay for stage-specific detection of *P. falciparum* parasites^12^, we were able to confirm the persistence of mature gametocytes upon AL treatment and efficient clearance with the addition of 0.75 mg/kg primaquine (Fig. 1A and S2). Surprisingly, however, we also observed the presence of RNA transcripts suggestive of ring stage parasites at a prevalence of >25% until day 14 in both treatment arms (Fig. 1B). Estimates of parasite numbers using regression analysis based on data points with both microscopic and qRT-PCR detection of parasite stages (Fig. S1A) demonstrated reduction of the concentration of ring stages by >98% within the first 3 days of treatment but persistence at low levels until day 14 (Fig. 2B). Differential reduction in ring *versus* mature gametocyte loads upon treatment (Fig. 2A,B) resulted in an increase in the fraction of all parasites that were mature gametocytes. This fraction rose from <0.4% on day 0 to 20% on day 3 in the AL arm, and from 0.1 to 8.1% in the same time frame in the AL-PQ arm (Fig. 2C). We have previously demonstrated that the mature gametocyte marker is not expressed in ring stage parasites^12^, and although the ring stage marker is not exclusively expressed in the ring stage, we performed cellular sensitivity analysis across the parasite cycle for the ring stage marker, and showed that the observed signal of this marker could not originate from mature gametocytes in the patient samples (Fig. S1B). This hypothesis is supported by the lack of any significant correlation between the prevalence of ring stage marker and either *PF3D7_1438800* or *Pfs25* (from the QT-NASBA data) across patient samples and treatment arms. In contrast the two different gametocyte markers are highly correlated in the AL group (p-value = 2.2 × 10^−14^) and in the AL-PQ group (p-value = 3.2 × 10^−5^), and in the subset of persisting parasites in both groups (p-value = 1.1 × 10^−18^), using a partial correlation test controlling for the time of sampling. Together these data suggest the existence of low-level persistent ring stage parasites upon AL treatment, alone or in combination with primaquine, among African malaria patients from Uganda.

### Genotypic analysis of persisting ring stage parasites

**Figure 3:**
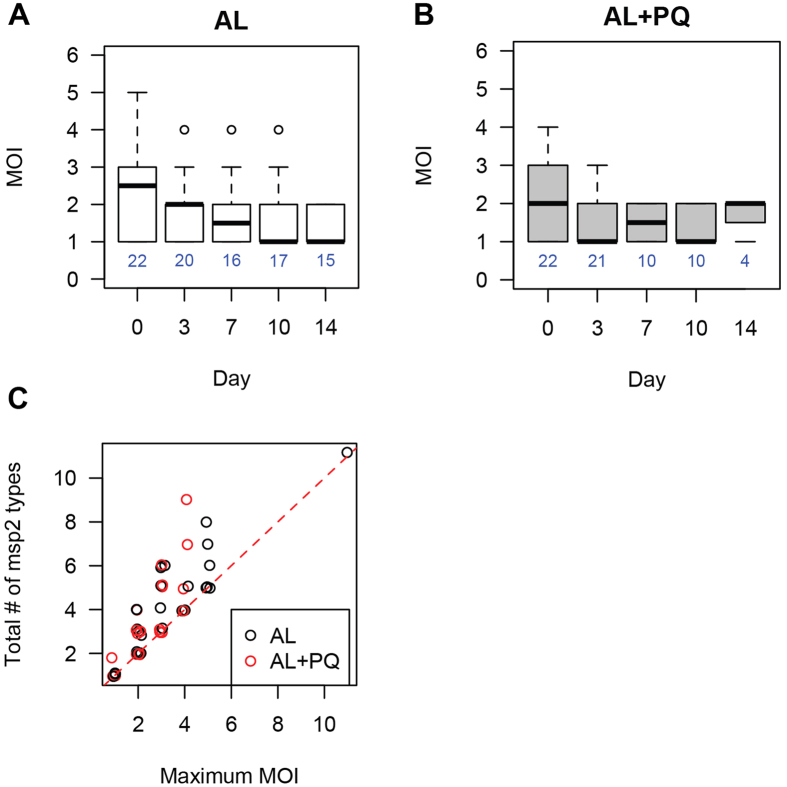
MSP2 genotyping to determine multiplicity of infection. **(A,B)** MOI decreases after treatment in both groups (Mann-Whitney test, *p*-values = 0.0016 [AL] and 0.0081 [AL + PQ]). Instead of having one dominant genotype, multiple genotypes are circulating on later days in some samples. One patient sample has MOI = 11 on day 0 in the AL group and is not shown in the figure. **(C)** Total number of *msp2* types is greater than maximum MOI in some patients, indicating that new genotypes emerge later in the patients.

**Figure 4:**
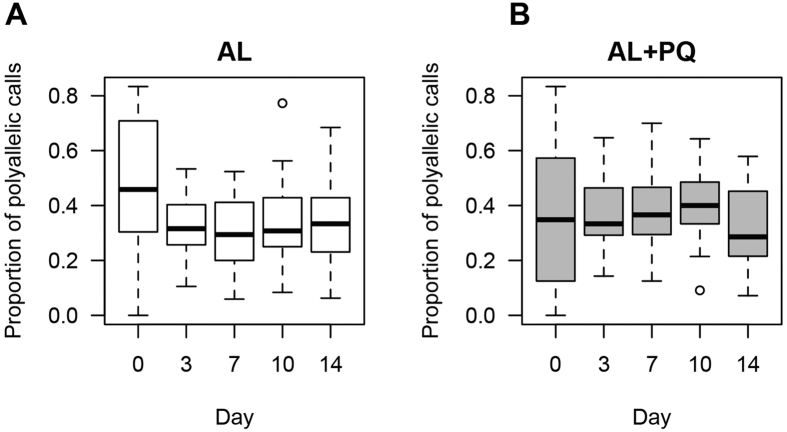
Molecular barcode analysis to determine multiplicity of infection. **(A,B)** Highly polymorphic molecular barcodes suggest that multiple genotypes are circulating both before and after treatment.

The observed presence of a low level reservoir of circulating ring stage parasites at day 14 demonstrated that these parasites persisted after a fully observed curative dose of AL. To determine possible selection of parasite subpopulations upon treatment we performed *msp2* genotyping (Fig. 3). We found that multiplicity of infection (MOI) decreased significantly in both the AL and the AL-PQ arms between day 0 and day 3 (Mann-Whitney Test, *p*-value = 0.05 and 0.02, respectively), however many of them remained >1 (Fig. 3A,B). Interestingly, a subset of *msp2* genotypes was significantly enriched in the persistent parasite population (Table S1). We also found that in several children the total number of *msp2* genotypes detected during follow-up was greater than the maximum MOI for any given day (Fig. 3C), suggesting some dynamics in the composition of the parasite population at later days post treatment. In 88.2% (16/18) of patients with successful *msp2* genotyping of persisting parasites on day 14 and all preceding days, the parasite genotypes on day 14 were also detected on preceding days. This indicates that re-infections were uncommon during the first 14 days of follow-up^14^ and the vast majority of parasites detected on day 14 were already present at enrollment. Similar analyses using a recently developed molecular barcode tool^15^ found a high proportion of polyallelic calls in the barcode (Fig. 4A,B) and that the major barcode alleles changed across the time of infection, suggesting that multiple clones are circulating in the patients and that the dominant genotype can fluctuate over time.

Residual ring stage parasitemia by day 3 was not significantly associated with patient age (medians are 4 [cleared] and 4.5 [residual], Mann-Whitney Test *p*-value = 0.70), enrollment parasite density (medians are 23,950 [cleared] and 26,670 [residual], Mann-Whitney Test *p*-value = 0.45) or any other patient or infection characteristics (Table 1). Similarly, residual ring stage parasitemia by day 14 was not associated with these patient or infection characteristics (Table S2). The proportion of individuals with gametocytes by microscopy, QT-NASBA or multiplexed qRT-PCR, at day 0 was not associated with residual ring stage parasitemia (Fisher’s Exact Test, *p*-values are 0.70 [microscopy], 0.61 [QT-NASBA], and 0.44 [qRT-PCR]).

## Discussion

Using a multiplexed qRT-PCR assay for stage-specific detection of *P. falciparum* mRNA transcripts, we provide evidence of persisting submicroscopic densities of ring stage parasites after successful treatment with either AL alone or plus primaquine. While parasite densities and the estimated number of clones in infections were reduced in the first 3 days after treatment^9^, more than 25% of the patients harbored low-density parasite populations based on stage-specific transcript quantification, that were frequently clonally complex and persisted for at least 14 days after initiation of treatment. Transcripts of our ring marker disappear after radical cure of experimental infections in human volunteers; when subcurative doses are administered and recrudescent infection occurs, ring marker transcripts reappear in the blood coincidentally with recrudescence (James McCarthy, personal communication). Although the dynamics of transcript clearance may be different in naturally exposed malaria-infected individuals who have clonally complex infections, non-synchronous infections and pre-exiting immunity, our findings suggest the persistence of very low concentrations of ring-stage parasites after apparently successful ACT treatment.

The role of persisting submicroscopic infections in determining treatment outcome, transmission dynamics and possibly drug resistance remains to be established. Previous studies on continued PCR positivity after ACT treatment were unable to reliably differentiate between residual circulating DNA molecules or gametocytes^8^,^9^,^16^,^17^. Our analysis based on stage specific mRNA transcripts, overcomes the uncertainties of DNA-based assays. Although our findings fall short of conclusively showing that RNA transcripts represent viable parasites, they may shed new light on parasite clearance dynamics after ACT treatment. Parasite carriage has been reported to persist for several months in a fraction of chloroquine-treated patients^18^,^19^. This chronic carriage is predominantly submicrosopic, only rarely being detectable by microscopy, and associated with continued gametocyte production^18^,^19^. It is conceivable that also after AL a fraction of patients may continue to harbor viable parasites that persist as chronic infections. In our study, the apparent persistence of ring stage parasites was not associated with gametocyte carriage or later recurrent parasite carriage, as recrudescence was only detected in 3.7% of patients^13^. We therefore did not observe direct consequences of residual ring stage parasitemia in terms of clinical response or transmission potential during the 28 days of follow-up. This also means that we did not provide conclusive evidence on the viability of the parasite populations that we detect based on ring-stage RNA. Studies that involve monitoring parasite populations for a longer period of follow-up or studies that attempt to culture very low parasite densities that persist after treatment may provide more definitive evidence for the viability and relevance of the parasite populations we detected. Our findings may also reflect a normal but previously unknown process of slow clearing, submicroscopic parasite populations that is specific to ACT treatment.

We hypothesized that increasing age, being an indicator of higher cumulative malaria exposure and thus immunity, would be associated with faster clearance of infections^20^. However, we observed persistence of rings among all ages (1 to 10 years), and there was no significant difference in age between patients with cleared and residual parasitemia. We observed that the number of rings at day 0 decreases with age, while neither the number of mature gametocytes nor MOI at day 0 change with age (Fig. S3). These observations suggest that age and therefore increasing exposure protects from high (asexual) parasite burden but not from gametocyte burden. Hence, the proportion of the total parasite population that represents mature gametocytes increases with age (irrespective of treatment), consistent with previous studies using microscopy or QT-NASBA data^21^,^22^.

The estimated ring stage parasite concentrations were very low beyond day 3 after initiation of treatment, with values below 2% of the original parasite density^8^. The precision of individual parasite densities is limited at these low concentrations, illustrated by our mean estimated densities of ~200 parasites/μl at day 3, which, although not detected by microscopy in our study, should be detectable by an expert microscopist. A better quantification of low parasite densities requires model optimization that would benefit from a larger number of natural low-density infections with asexual parasite quantification by qRT-PCR and microscopy to take into account possible variation in transcript numbers between parasite strains.

The reason for the persistence of parasite transcripts that is suggestive of ring-stage persistence, after apparently successful treatment is unknown. Low levels of persisting parasites might originate from dormant parasite populations or a sequestered reservoir, might represent remaining parasites during therapy due to therapeutic pharmacokinetic properties, or might be associated with resistance to drugs. We observed no evidence of an association with drug resistance, parasitological and clinical cure rates by microscopy were excellent^13^, as is reported elsewhere in Uganda^23^,^24^. If the persistence would be due to drug resistance, only drug-resistant lineages could persist and we expect to observe low MOI and similar parasites that are persisting. However, although MOI decreased after treatment, many MOIs were greater than one (Fig. 3A,B), the major barcode changed through time, and persisting parasites have very diverse *msp2* types (although two *msp2* types are enriched among persisting parasite populations). The detection of *msp2* genotypes at individual time points is imperfect and influenced by parasite densities, sequestration patterns and differences in amplification efficiency of smaller and larger *msp2* alleles^25^. Although this may have influenced the accuracy of MOI estimates at individual time points, it is unlikely to have affected comparisons between treatment arms and a decline in MOI over time, or persistence of certain genotypes. Overall, our findings suggest that ring persistence is not caused by drug resistance selection on a particular genetic background. *In vitro* studies have revealed that parasites may be able to tolerate artemisinin treatment by entering a temporarily growth-arrested state of dormancy^10^ from which a fraction may recover. If the persisting ring stage parasites in our population reflect such dormant parasite populations, re-treatment of individuals with the same drug may be equally efficacious^26^ and ring stage parasite persistence should not be seen as an indicator of parasite resistance or clinically relevant altered clearance dynamics. Persisting ring stage parasites may also originate from a sequestered reservoir that is partially protected from drug treatment. Recent identification of the human bone marrow as a reservoir for parasite sequestration and gametocyte development^27^ supports this hypothesis and would explain our finding of newly appearing genotypes during follow up; this may also be associated with the imperfect detectability of individual parasite clones^28^(Fig. 2A). We observed that the majority of these new clones appeared in the first week after initiation of treatment, reinforcing previous observations that re-infections are unlikely within the first 14 days after AL^14^, and suggesting that they are an unlikely explanation for the observed long persistence of ring stage parasites in our study.

In summary, we present preliminary evidence for persisting submicroscopic parasite populations that comprise ring stage parasites and gametocytes after treatment with AL or AL-PQ, treatment combinations that were efficacious in preventing recurrent parasite carriage by microscopy for at least 28 days. While the viability of these parasite populations remains to be established and we observed no evidence that parasite populations have no immediate clinical consequences for the treated patients, our findings are relevant for the interpretation of parasite clearance and transmission dynamics. Future *in vivo* studies are needed to confirm that the persistence of transcripts of ring stage parasites indicate viable parasite populations. These studies should be designed to determine whether the phenomenon of ring stage persistence is a characteristic of ACT treatment and allow for the assessment of late recrudescent infections and/or *de novo* generation of gametocytes from low levels of persisting parasite populations.

## Methods

### Ethics statement

All experiments were performed in accordance with relevant guidelines and regulations. Specifically samples for this study were derived from a previously published randomized, double blind, placebo-controlled trial with four treatment arms^13^. This study was approved by Makerere University School of Medicine (2011–210), the Uganda National Council of Science and Technology (HS1056), and the London School of Hygiene and Tropical Medicine (5987) and was registered at ClinicalTrials.gov (NCT01365598). Informed consent was obtained from all subjects, and all samples were made anonymous for analysis.

### Study cohort

Children aged 1–10 years with uncomplicated *P. falciparum* mono-infection with a parasite density lower than 500,000 parasites per *μ*L, normal G6PD enzyme function and hemoglobin concentration ≥ 80 g/L were randomized to treatment with AL alone (AL group) or in combination with 0.1, 0.4 or 0.75 mg/kg primaquine (AL-PQ group). For the current analysis, we selected 37 children from the AL arm and 33 children from the AL-PQ arm with a dose of 0.75 mg/kg primaquine, using random tables generated in Stata (version 12.0). Children were followed for 28 days for safety parameters, recrudescence or re-infection. Blood samples were obtained by finger prick on days 0, 3, 7, 10 and 14 after initiation of treatment, and immediately transferred to L6 buffer (Severn Biotech Limited, Kidderminster, UK) and stored at −80 °C until extraction. Nucleic acid material was extracted from 50 μL aliquots using Total Nucleic Acid Isolation Kits–High Performance (Roche Applied Science, Mannheim, Germany) and a MagNA Pure LC automated extractor (Roche Applied Science).

### Molecular detection of parasite stages

The patient’s parasite burden was quantified using microscopy, gametocyte detection by *Pfs25* mRNA quantitative nucleic acid sequence based amplification^13^ and with a highly sensitive stage-specific qRT-PCR assay, as described previously^12^. The latter assay combines stage-specific markers to quantify the relative abundance of asexual and sexual *P. falciparum* parasites from patient samples using probe-based qRT-PCR. Reverse transcription was performed from extracted nuclear acid samples using SuperScript III First-Strand Synthesis System (Life Technologies, Carlsbad, CA). Custom primers (Integrated DNA Technologies, Coralville, IA) and Taqman probes (Life Technologies) were used at 900 nM and 250 nM final concentrations, respectively, to measure the transcript levels of *PF3D7_0501300* (previous accession number *PFE0065w*; for ring stage parasites), *PF3D7_1438800* (*PF14_0367*; for mature gametocyte parasites) and *PF3D7_1120200* (*PF11_0209*; as a constitutive parasite marker) on a Viia7 qRT-PCR machine (Life Technologies).

A linear regression model was developed to estimate the density of circulating ring and mature gametocyte parasites. We used the patient’s patent parasitemia (i.e., density of ring and mature gametocyte stages) obtained using Giemsa stained microscopy slide to define the relationship between parasite density and CT values from qRT-PCR (ring and mature gametocyte marker, respectively) by regression analysis. The regression between microscopy and qRT-PCR was then used to infer parasite density in the remainder of the data points with sub-patent parasite levels (Fig. S1A). Multiple linear regressions were used to test the difference in parasite density in the AL and the AL + PQ groups while controlling for the day on which the samples were collected.

To determine the sensitivity of our ring marker detection by qRT-PCR assay, we performed a cellular dilution experiment under controlled *in vitro* conditions with stage-specific populations of *P. falciparum* strain 3D7 (Fig. S1B). Parasites were cultured in human type O+ red blood cells (Research Blood Components, Boston, MA) diluted to 4% hematocrit in RPMI-1640 medium (Sigma-Aldrich, St. Louis MO) supplemented with 10% human serum (Interstate Blood Bank, Memphis, TN), 25 mM HEPES (EMD Biosciences), sodium bicarbonate (Sigma), and hypoxanthine (Sigma), as described previously^29^. Parasite cultures were maintained at 37 °C in an atmosphere of 1% O_2_ and 5% CO, 94% N_2_. The parasites were synchronized with 5% D-sorbitol, and time points were collected for pure stage-specific populations at 8, 16, 24, 32, 40 hours post invasion for asexual parasite stages, and days 2, 4, and 7 post gametocyte induction using partially spent (“conditioned”) medium for sexual parasite stages^30^,^31^. Serial dilutions of purified red blood cells (RBC) infected synchronized populations of the above parasite stages were made into uninfected RBCs, and RNA was extracted using Trizol (Life Technologies) and RNeasy (Qiagen, Valencia, CA) for subsequent cDNA synthesis and qRT-PCR as described above.

### Multiplicity of infection and genotyping analysis

DNA from all time points was tested for clonal complexity based on genotyping for the polymorphic marker gene merozoite surface protein 2 (*msp2*) using capillary electrophoresis for fragment sizing^32^ following chelex-saponin extraction from filter papers (Whatman 3 MM, Maidstone, UK). Results were analyzed with Peak Scanner version 1.0 (Applied Biosystems) with discrete peaks being interpreted as unique clones, after adjustment for background levels per experiment. Nucleic acids from the whole blood samples in L6 buffer that were extracted by MagNA Pure LC automated extractor, as described above, were further analyzed using previously described molecular barcode methods^15^. Briefly, 1 μL of each extracted sample was preamplified^33^ in 20 μL reactions using the TaqMan Preamp Master Mix for 14 cycles according to manufacturer’s directions (Applied Biosystems) using 0.2× pooled TaqMan barcoding assays. Samples were diluted 1:20 and 5 μL each preamplified product used for the molecular barcode. TaqMan barcoding assays were run on a ViiA system (Applied Biosystems) and genotypes called using the pre-installed analysis software.

## Additional Information

**How to cite this article**: Chang, H.-H. *et al*. Persistence of *plasmodium* *falciparum* parasitemia after artemisinin combination therapy: evidence from a randomized trial in Uganda. *Sci. Rep.* **6**, 26330; doi: 10.1038/srep26330 (2016).

## Supplementary Material

Supplementary Information

## Figures and Tables

**Table 1:** Characteristics of cleared and residual parasitemia*.

	**Cleared parasitemia**	**Residual parasitemia**	***p*****-value**
Number of patients (day 3)	9	61	
Number of patients (day 14)	55	15	
Age	4	4.5	0.70
Gender (male:female)	5:4	27:34	0.72
Asexual parasite densityϕ by microscopy at enrollment	23950	26670	0.45
Gametocyte prevalence by microscopy at enrollment	50%	37%	0.70
Gametocyte prevalence by microscopy at day 7	0	7.84%	1
Treatment, % AL + PQ treated	44%	49%	1
MOI at enrollment	1.5	2	0.38

^ϕ^Unit of parasite density is the number of parasites per *μ*l.

^*^Medians are shown.
