# Supplementary material for: Persistence of Plasmodium falciparum parasitemia after artemisinin combination therapy: evidence from a randomized trial in Uganda
Source: Sci Rep. 2016 May 20;6:26330. doi: 10.1038/srep26330 (PMC4873826; doi:10.1038/srep26330)
Supplement: Supplementary Information [file srep26330-s1.pdf]

Supplemental Data

Persistence of *Plasmodium falciparum* parasitemia after artemisinin combination therapy: evidence from a randomized trial in Uganda

Hsiao-Han Chang[1]*, Elamaran Meibalan[2]*, Justin Zelin[2]*, Rachel Daniels[2,3]*, Alice C. Eziefula[4], Evan C. Meyer[2], Fitsum Tadesse[4], Lynn Grignard[4], Regina Joice[2], Chris Drakeley[4], Dyann F. Wirth[2,3], Sarah K. Volkman[2,3,5], Caroline Buckee[1], Teun Bousema[4,6]^, Matthias Marti[2]^

[1]Center for Communicable Disease Dynamics, Department of Epidemiology and [2]Department of Immunology and Infectious Diseases, Harvard T.H. Chan School of Public Health, Boston, USA

[3]Broad Institute of MIT and Harvard, Cambridge, USA

[4]Immunology and Infection Department, London School of Hygiene and Tropical Medicine, London, UK

[5]Simmons College, Boston, USA

[6]Institute for Molecular Life Sciences, Radboud University, Nijmegen, The Netherlands

*These authors contributed equally to this study

Figure S1

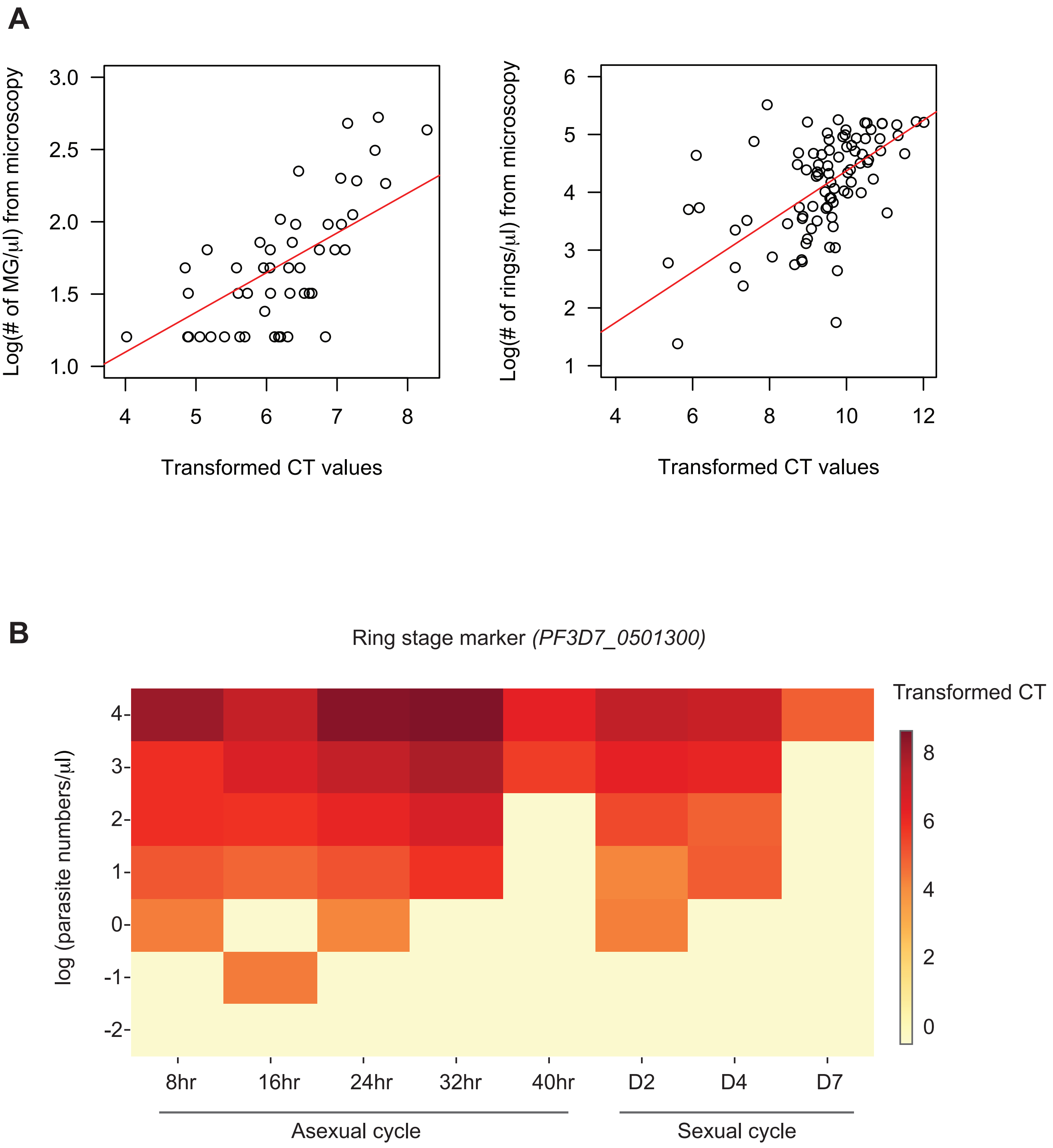
A
Log(# of MG/μl) from microscopy
3.0
2.5
2.0
1.5
1.0
4
5
6
7
8
Transformed CT values
Log(# of rings/μl) from microscopy
6
5
4
3
2
1
4
6
8
10
12
Transformed CT values
B
Ring stage marker (PF3D7_0501300)
log (parasite numbers/μl)
4
3
2
1
0
-1
-2
8hr
16hr
24hr
32hr
40hr
D2
D4
D7
Asexual cycle
Sexual cycle
Transformed CT
8
6
4
2
0

Figure S2

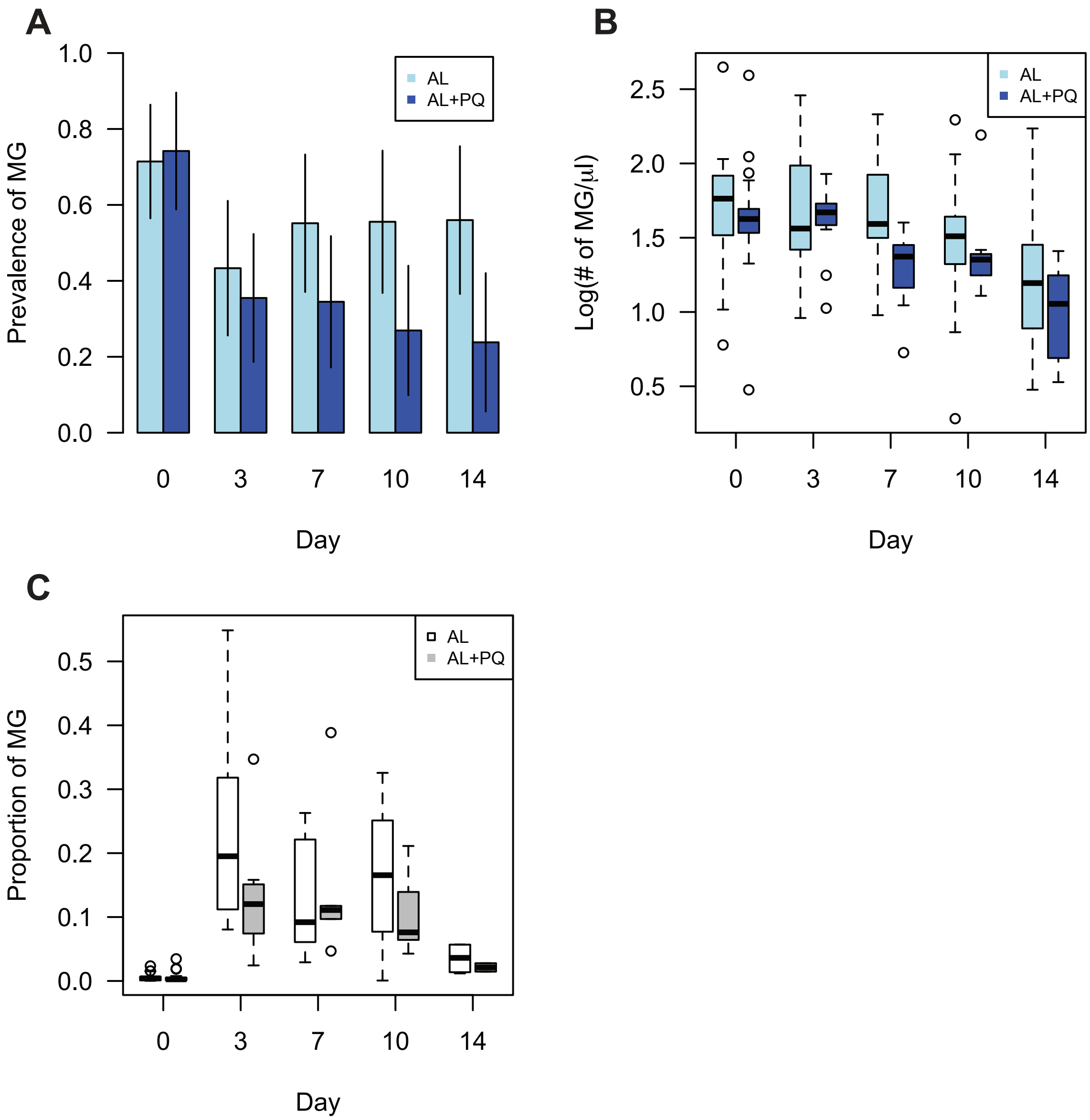
A
B
C
Prevalence of MG
Log(# of MG/µl)
Proportion of MG
Day
AL
AL+PQ
0
3
7
10
14

Figure S3

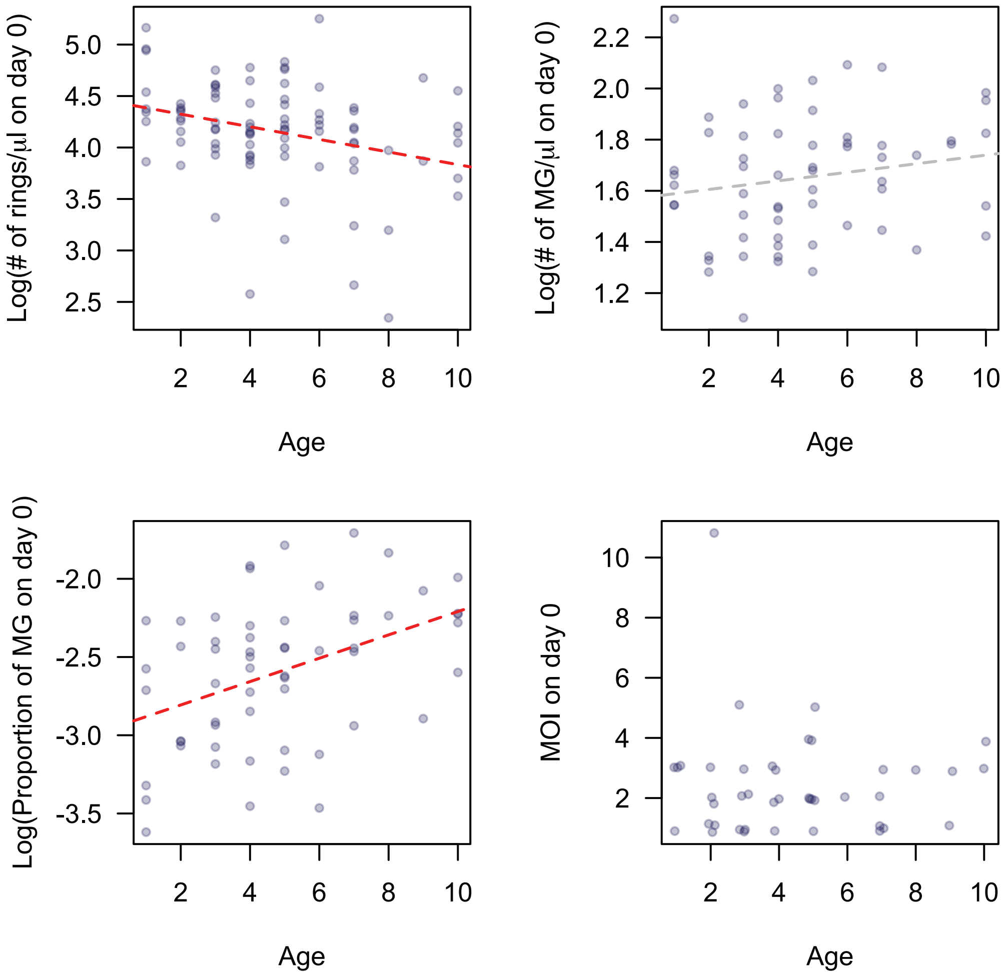
Log(# of rings/μl on day 0)
5.0
4.5
4.0
3.5
3.0
2.5
Log(# of MG/μl on day 0)
2.2
2.0
1.8
1.6
1.4
1.2
Log(Proportion of MG on day 0)
-2.0
-2.5
-3.0
-3.5
MOI on day 0
10
8
6
4
2
2
4
6
8
10
Age

Table S1. Persisting parasites are more likely to have certain *msp2* types

Persisting parasite	Persisting on day	# of persisters with this type	# of persisters without this type	# of non-persisters with this type	# of non-persisters without this type	Fisher's exact test *p*-value
			**3D7_275**			
Ring	10	6	31	0	61	0.0022
Ring	14	5	14	1	78	0.0009
Gametocyte	3	6	37	0	55	0.00579
Gametocyte	10	4	23	2	69	0.0472
			**Fc27_400**			
Ring	7	8	36	2	52	0.039
Ring	10	8	29	2	59	0.0056
Ring	14	5	14	5	74	0.022

Table S2. Characteristics of day 14 cleared and residual parasitemia*

	Cleared parasitemia	Residual parasitemia	*p*-value
Number of patients (day 14)	55	15	
Age	4	5	0.69
Gender (male:female)	26:29	6:9	0.77
Asexual parasite density[ϕ] by microscopy at enrolment median	24500	48530	0.49
Gametocyte prevalence by microscopy at enrollment	38%	40%	1
Gametocyte prevalence by microscopy at day 7	6.98%	7.14%	1
Treatment, % AL+PQ treated	51%	40%	0.56
MOI at enrollment	2	2	0.46

[ϕ] Unit of parasite density is the number of parasites per μl.

* Medians are shown if not mentioned.

Supplemental figure legends

Figure S1. (A) Regression for ring and mature gametocyte stages based on the correlation between microscopy and qRT-PCR. The intercept was forced to be zero because parasite density is expected to be zero when transformed CT value is 0. Transformed CT value (CT*) = $\log(2^{-CT}) + 17$. The fitted function for ring density (per μl) = $10^{CT^* \times 0.4371}$ and the fitted function for MG density (per μl) = $10^{CT^* \times 0.2746}$. The *p*-values for the slopes are both smaller than 2×10^{-16}. The correlation coefficients *r* between microscopy and qRT-PCR are 0.71 (*p*-value = 1.4×10^{-8}) and 0.53 (*p*-value = 1.1×10^{-7}), respectively. **(B) Sensitivity of ring stage marker across the *P. falciparum* red blood cell cycle.** Ring marker can detect up to 0.1 parasite/μl (16 hr time point). Ring marker transcript in mature gametocytes is only detected when numbers are >1000 parasites/μl (day 7 time point). Notably, gametocyte numbers in the patients are <100 gametocytes/μl.

Figure S2. Changes in asexual and sexual stage parasite prevalence over time based on Pfs25 QT-NASBA data [13]. **(A)** The prevalence of mature gametocytes (MG) decreases more rapidly in the AL-primaquine (AL + PQ) group (the slope of simple linear regression = −0.0048 (95% CI = [−0.024, 0.015]) for the AL group and −0.031 [95% CI = [−0.053, −0.0076] for the AL+PQ group). **(B)** The number of mature gametocytes after day 3 is significantly smaller in the AL+PQ group (*p*-value = 0.023). **(C)** The proportion of the total parasite population that is mature gametocytes increases during follow-up (Mann-Whitney test, *p*-values = 1.93×10^{-11} [AL] and 9.23×10^{-12} [AL+PQ]). There is no patient in the AL+PQ group having both rings and MG data on day 14.

Figure S3. Age versus parasite density and MOI. Ring density decreases with age (Pearson's correlation test between log of the number of rings and age, $r = -0.30$, p-value = 0.0049) while mature gametocyte density (Pearson's correlation test between log of the number of MG and age, $r=0.18$, p-value = 0.17) and MOI (Spearman's rank correlation test, p-value = 0.71) do not, leading to the increase in the proportion of mature gametocytes with age.
